# Increased risk of venous thrombosis by AB alleles of the ABO blood group and Factor V Leiden in a Brazilian population

**DOI:** 10.1590/S1415-47572009000200010

**Published:** 2009-06-01

**Authors:** Magaly B. P. L. V. Lima, Aldemir Branco de Oliveira-Filho, Júlia F. Campos, Fárida C. B. C. Melo, Washington Batista das Neves, Raul Antônio Morais Melo, José Alexandre Rodrigues Lemos

**Affiliations:** 1 Laboratório de Biologia Celular e Molecular, Fundação Centro de Hemoterapia e Hematologia do Pará, Belém, PABrazil; 2 Laboratório de Biologia Molecular, Fundação Centro de Hematologia e Hemoterapia de Pernambuco, Recife, PEBrazil; 3 Instituto de Ciências Biológicas, Universidade Federal do Pará, Belém, PABrazil

**Keywords:** ABO blood group, Factor V Leiden, venous thrombosis

## Abstract

Most cases of a predisposition to venous thrombosis are caused by resistance to activated protein C, associated in 95% of cases with the Factor V Leiden allele (FVL or R506Q). Several recent studies report a further increased risk of thrombosis by an association between the AB alleles of the ABO blood group and Factor V Leiden. The present study investigated this association with deep vein thrombosis (DVT) in individuals treated at the *Hemocentro de Pernambuco* in northeastern Brazil. A case-control comparison showed a significant risk of thrombosis in the presence of Factor V Leiden (OR = 10.1), which was approximately doubled when the AB alleles of the ABO blood group were present as well (OR = 22.3). These results confirm that the increased risk of deep vein thrombosis in the combined presence of AB alleles and Factor V Leiden is also applicable to the Brazilian population suggesting that ABO blood group typing should be routinely added to FVL in studies involving thrombosis.

Deep vein thrombosis (DVT) is one of the main clinical manifestations of venous thromboembolism (VTE). DVT is a multi-factorial disorder caused by genetic and/or acquired abnormalities of the hemostatic mechanism, resulting in hypercoagulation (Dahlbäck, 2003; Dahlbäck and Villoutrex, 2003). Hereditary thrombophilia is responsible for most cases of predisposition to VTE and the greatest prevalence is of a resistance to activated protein C (Svensson and Dahlbäck, 1994), caused in most cases by a point mutation in the gene of the coagulation factor V (FV), known as Factor V Leiden (FVL), FV: R506Q or FV: G1691A. (Bertina *et al.*, 1994). FV is a plasma glycoprotein of 330 kDa, formed by 2196 amino acids and coded by a gene with 25 exons located in chromosome region 1q21-25 (Wang *et al.*, 1988; Cripe *et al.*, 1992). In its activated form (FVa), it participates in the prothrombinase complex of the coagulation cascade as an essential co-factor to factor Xa in thrombin genesis. FV is also a proteolytic target for activated protein C (APC), which exercises its anti-coagulant function by deactivating it. The deactivated FV and protein S act as co-factors of APC, deactivating the factor VIII (FVIII) molecule (Dahlbäck and Villoutrex, 2003). The R506Q mutation occurs in exon 10 of the FV gene and is characterized by a G → A transition at nucleotide 1691, which results in a change from Arginine to Glutamine (Arg → Gln) at position 506 in the amino acid sequence (Bertina *et al.*, 1994). The main consequence of this mutation is the loss of one of the cleavage sites, which makes FVa partially resistant to the proteolytic degradation of APC. This flaw in the anticoagulation mechanism mainly results in high FVIII and trombin levels, thereby increasing the risk of VTE due to the installation of a state of hypercoagulability (Franco and Reitsma, 2001).

The AB alleles of the ABO blood group, high FVIII levels and von Willebrand factor (vWF) have been associated to a risk of venous thrombosis (Jick *et al.*, 1969; Koster *et al.*, 1995; Tirado *et al.*, 2005). A number of reports reveal that individuals with the AB genotype have higher FVIII and vWF levels than those with the O genotype (Preston and Barr, 1964; Orstavick *et al.*, 1985; Gill *et al.*, 1987; Kamphuisen *et al.*, 1998). Studies have also reported that the association between the R506Q allele and AB alleles increases the risk of thrombosis (Robert *et al.*, 2000; Morelli *et al.*, 2005; Biron-Adréani *et al.*, 2006; Ohira *et al.*, 2007). The present study investigated the relationship between DVT and the association of AB genotypes and the R506Q allele in the population of the state of Pernambuco in northeastern Brazil.

Sixty-five patients participated in the study (47 females and 18 males) with a mean age of 34 years (range 6-67 years) and no familial relationships to one another. All patients had a history of DVT and were treated at the outpatient clinic of the Fundação HEMOPE (Pernambuco, Brazil) between 2001 and 2006. An additional 51 individuals from the same state and with no history of DVT were selected for the control group, which was paired for gender and age group and had no family relationships to one another or to the patients. All samples were collected following the informed consent of the participants.

Peripheral venous blood samples were collected in a sterile *vacutainer* tube containing EDTA as the anticoagulant. Genomic DNA was extracted and purified from leukocytes using the GFX genomic DNA purification kit (*Amershan Pharmacia Biotech*), following the manufacturer's instructions. Genotyping of the ABO system and detection of the R506Q allele in the patients and controls were performed through real-time polymerase chain reaction/allelic discrimination assay using the ABI PRISM 7000 Sequence Detection System (SDS). For the amplification reactions, the Taqman Universal PCR Master Mix (Applied Biosystems, Foster City, CA) set of reagents was used together with a set of primers and specific probes for the differentiation between the O and A or B genotypes and differentiation between wild alleles and R506Q, designed and synthesized by the Assays-by-Design service (Part Number 4331349) from the sequences of interest sent to Applied Biosystems. In the text and Tables, the genotype designation O refers to absence of any A or B alleles at the ABO locus and AB refers to any of the following: AA, AO, AB, BB, or BO.

Following amplification, the fragments were discriminated by fluorescent data analysis through the software program (version 1.1) on the ABI PRISM 7000 SDS equipment. Statistical analysis was performed by univariate analysis, using the Bioestat 4.0 program (Ayres *et al.*, 2005). Confidence limits were set at 95% and exact p values determined using Pearson's Chi square.

The frequencies of patients and controls, ABO and FVL genotypes and Odds Ratios of increased risk of DVT are displayed in Table 1.

We have assumed that all or the majority of FVL (+) individuals were heterozygotes and carried only a single mutant allele, which is a reasonable assumption given the allele frequency of between 0.02-0.05 seen in many populations. In the patient group, 29.3% had the R506Q allele, whereas only 3.9% in the control group. This led to a highly significant risk of DVT (OR = 10.1; 95%CI = 2.23-45.9; p = 0.0004) which was higher than that described in the study by Morelli *et al.* (2005), which was 7.9. This risk was increased further in individuals with the AB/FVL(+) genotype combination (OR = 22.3; 95%CI = 2.68-185.7; p = 0.0002) These results are compatible with all previous investigations on the combined additive contribution of ABO and FVL genotypes to DVT. The results of previously published studies are summarized in Table 2. All studies demonstrate the added risk of AB alleles which approximately doubles the risk imparted by FVL. The differences in ORs between studies are probably explicable on small sample sizes and ethnic or chance differences in observed ABO and FVL allele frequencies. The exact mechanism through which the AB alleles influence the risk of thrombosis is not yet well clarified. However, evidence suggests that it could be explained by the effect of the ABO locus on the rise in plasma levels of FVIII (Morelli *et al.*, 2005), likely due to a lower catabolism of the vWF (Gill *et al.*, 1987). The presence of oligosaccharides with A, B and O structures in the human vWF molecule support this hypothesis (Sodetz *et al.*, 1979; Matsui *et al.*, 1992). In individuals with the FVL who have the AB genotype, the weak deactivation of FVIII in combination with high levels of this factor through the influence of the ABO genotype are thought to result in an exponential increase in the risk of developing VTE (Morelli *et al.*, 2005; Váradi *et al.*, 1996).

The present study confirms that the association of a combined increased risk of AB alleles and FVL to deep vein thrombosis is also applicable to the population of North Eastern Brazil. We propose that ABO genotyping should be carried out routinely in combination with FVL to evaluate risk for DVT. It would also be of great interest to study whether ABO alleles also have an additive affect in other situations, such as spontaneous abortion or reduced menopausal age, where FVL has also been claimed to play a role.

## Figures and Tables

**Table 1:** Combined risk of thrombosis for ABO and FVL genotypes.

Genotypes	N. of patients (%) n = 65	N. of controls (%) n = 51	OR exact p value	CI (95%)
O	18 (27.7)	22 (43.1)	1*	
AB	47 (72.3)	29 (56.9)	1.98	0.91-4.30
			p = 0.42	

FVL (-)	46 (70.7)	49 (96.0)	1*	
FVL (+)	19 (29.2)	2 (3.9)	10.11	2.23-45.88
			p = 0.0004	

O and FVL (–)	16 (24.6)	21 (41.2)	1*	
O and FVL (+)	2 (3.1)	1 (1.96)	2.62	0.21-31.5
AB and FVL (–)	30 (46.1)	28 (54.9)	1.40	0.61-3.22
AB and FVL (+)	17 (26.2)	1 (1.96)	22.3	2.68-185.7
			p = .0002	

FVL (+) = assumed heterozygotes ; FVL (–) = wild type homozygotes.*Reference category. OR = odds ratio. CI (95%) = 95% confidence interval.

**Table 2:** Different risks of DVT in the combined presence of AB alleles and FVL allele

Study	Population	Patients	Controls	OR
Present	Brazilian	65	51	22.3
Robert *et al.*(2000)	French	147	32*	3.9
Morelli *et al.*(2005)	Dutch	471	471	23.2
Ohira *et al.*(2007)	African Americans and whites	487	1006	6.77

OR = odds ratio, *FVL heterozygotes.
